# Reimagining Colorectal Cancer Screening: Innovations and Challenges with Dr. Aasma Shaukat

**DOI:** 10.3390/cancers16101898

**Published:** 2024-05-16

**Authors:** Viviana Cortiana, Muskan Joshi, Harshal Chorya, Harshitha Vallabhaneni, Shreevikaa Kannan, Helena S. Coloma, Chandler H. Park, Yan Leyfman

**Affiliations:** 1 Department of Medical and Surgical Sciences (DIMEC), University of Bologna, 40126 Bologna, Italy; 2 Tbilisi State Medical University, Tbilisi 0186, Georgia; 3 Medical College Baroda, Vadodara 390018, India; 4 Apollo Institute of Medical Sciences and Research, Hyderabad 517001, India; 5 Harvard University, Cambridge, MA 02138, USA; 6 Norton Cancer Institute, Louisville, KY 40202, USA; chandler.park@louisville.edu; 7 Icahn School of Medicine at Mount Sinai, New York, NY 10029, USA; yan.leyfman@mssm.edu

**Keywords:** colorectal cancer, screening, early detection, adherence, artificial intelligence, blood-based tests, epidemiology

## Abstract

**Simple Summary:**

Colorectal cancer (CRC) stands as the third most common cancer globally and the second leading cause of cancer-related deaths, imposing a substantial health burden. Recent studies highlight a concerning rise in CRC rates among individuals below 50 years old, prompting the American Cancer Society (ACS) to recommend screening starting at age 45 for average-risk individuals. Dr. Aasma Shaukat’s Keynote Conference stresses the urgent need for updated screening strategies to tackle suboptimal adherence rates and effectively manage the increasing burden of CRC. Lowering the adenoma detection screening age could aid in early identification of adenomas in asymptomatic younger patients, potentially reshaping disease epidemiology. Current screening options encompass stool-based tests like multitarget stool DNA (mtDNA) tests, fecal immunochemical testing (FIT), and imaging-based tests. Moreover, blood-based tests are emerging as promising tools for early CRC detection, utilizing innovative techniques and AI algorithms. Medicare mandates specific criteria for national coverage of blood-based tests. Ongoing clinical trials, such as Freenome, Guardant, and CancerSEEK, offer hope for further advancements in blood-based CRC screening. Despite breakthroughs, accessibility and affordability challenges persist. Adapting healthcare systems to accommodate changing CRC epidemiology is imperative. Lowering the screening age and integrating blood-based tests hold the potential to alleviate the CRC-related burden amidst evolving epidemiology.

**Abstract:**

Colorectal cancer (CRC) currently ranks as the third most common cancer and the second leading cause of cancer-related deaths worldwide, posing a significant global health burden to the population. Recent studies have reported the emergence of a new clinical picture of the disease, with a notable increase in CRC rates in younger populations of <50 years of age. The American Cancer Society (ACS) now recommends CRC screening starting at age 45 for average-risk individuals. Dr. Aasma Shaukat’s Keynote Conference highlights the critical need for updated screening strategies, with an emphasis on addressing the suboptimal adherence rates and the effective management of the growing burden of CRC. Lowering the adenoma detection screening age can facilitate early identification of adenomas in younger asymptomatic patients, altering the epidemiologic landscape. However, its implications may not be as profound unless a drastic shift in the age distribution of CRC is observed. Currently, various screening options are available in practice, including stool-based tests like multitarget stool DNA (mtDNA) tests, fecal immunochemical testing (FIT), and imaging-based tests. In addition to existing screening methods, blood-based tests are now emerging as promising tools for early CRC detection. These tests leverage innovative techniques along with AI and machine learning algorithms, aiding in tumor detection at a significantly earlier stage, which was not possible before. Medicare mandates specific criteria for national coverage of blood-based tests, including sensitivity ≥ 74%, specificity ≥ 90%, FDA approval, and inclusion in professional society guidelines. Ongoing clinical trials, such as Freenome, Guardant, and CancerSEEK, offer hope for further advancements in blood-based CRC screening. The development of multicancer early detection tests like GRAIL demonstrates a tremendous potential for detecting various solid tumors and hematologic malignancies. Despite these breakthroughs, the question of accessibility and affordability still stands. The ever-evolving landscape of CRC screening reflects the strength of the scientific field in light of an altered disease epidemiology. Lowering screening age along with the integration of blood-based tests with existing screening methods holds great potential in reducing the CRC-related burden. At the same time, it is increasingly important to address the challenges of adaptation of the healthcare system to this change in the epidemiologic paradigm.

## 1. Introduction

Colorectal cancer (CRC) represents a significant challenge in oncology, imposing a substantial global health burden as the third most common cancer worldwide and the second leading cause of cancer-related deaths [1]. Dr. Aasma Shaukat’s enlightening Keynote Conference, “Updates in Colorectal Cancer Screening”, provides insight into the latest advancements shaping this critical healthcare field [2]. CRC often develops slowly from precursor lesions like adenomatous polyps or sessile serrated lesions, offering an opportunity for early detection and intervention. However, despite the benefits of early screening, adherence rates remain suboptimal in many developed nations, necessitating more effective strategies [3].

Recent years have witnessed a notable shift in CRC screening guidelines due to emerging evidence of increased incidence among individuals under 50 [4]. Traditionally, screening began at age 50, but recent studies indicate that those born around 1990 face twice the risk of colon cancer and four times the risk of rectal cancer compared to those born around 1950, emphasizing the urgency of effective screening strategies for early detection and prevention [5]. However, challenges like increased healthcare system burden and accessibility to affordable screening must be addressed for successful implementation.

Various CRC screening options exist, including stool-based tests like multitarget stool DNA (mtDNA) tests, fecal immunochemical testing (FIT), blood-based tests like Septin-9, and imaging-based tests such as CT colonography (CTC) and colonoscopy [3]. The selection of a screening method may vary based on individual risk factors, resource availability, and patient preferences.

The American Cancer Society (ACS) now recommends CRC screening for average-risk individuals starting at age 45, employing a high-sensitivity stool-based test or visual examination [6]. Positive non-colonoscopy screening results should prompt follow-up colonoscopy. However, successful implementation will require efforts to raise awareness, improve accessibility, and ensure the affordability of screening options.

Colorectal cancer (CRC) has immense heterogeneity in its presentation and a patient’s prognosis varies greatly depending on the tumor’s stage at diagnosis. Screening is key to a better prognosis, as shown by Bretthauer et al. who report a decreased risk of CRC at 10 years in patients invited to undergo screening colonoscopy in their study than those who were assigned to no screening [7]. Tumor microenvironment (TME) in colorectal cancer (CRC) is another important factor, impacting the target drug therapy and cancer progression. The TME is made up of a variety of dynamic cell types, including regulatory T cells, tumor-associated macrophages, cancer-associated fibroblasts, and myeloid-derived suppressor cells, and extracellular factors that surround cancer cells and play structural and functional roles [2]. Due to this, the TME is a rich source for the identification of new drugs [8].

**Figure 1:**
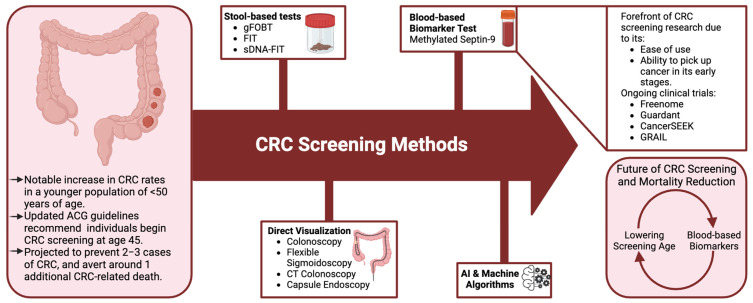
Present and future strategies of CRC screening methods. Created with www.biorender.com (accessed on 14 April 2024).

In conclusion, Dr. Shaukat’s presentation sheds light on the evolving CRC screening landscape, underscoring the significance of early detection in mitigating disease burden. This commentary aims to explore the implications of these changes, discussing the potential benefits and challenges associated with initiating CRC screening at an earlier age (Figure 1).

## 2. Adapting to Change: Evolving Strategies in Colorectal Cancer Screening Guidelines

The landscape of CRC screening has evolved significantly over the past decade, reflecting the dynamic nature of scientific understanding and the changing epidemiology of the disease. Until 2016, CRC screening guidelines in the United States recommended screening individuals between the ages of 50 and 75, without a clear preference for any specific test [9]. The guidelines from the U.S. Preventive Services Task Force (USPSTF) at that time detailed seven distinct tests within eight strategies, highlighting the absence of comparative effectiveness studies. The USPSTF recommends screening for CRC beginning at age 50 and continuing until age 75, using one of several screening modalities [10]. These include annual high-sensitivity Fecal Occult Blood Tests (FOBT), sigmoidoscopy every five years combined with high-sensitivity FOBT every three years, or colonoscopy every ten years. The American Cancer Society (ACS) recommends screening starting at age 45 for average-risk individuals. Other organizations, such as the American College of Gastroenterology (ACG) and the U.S. Multi-Society Task Force on Colorectal Cancer (MSTF), have similar recommendations for CRC screening. There are other screening guidelines also; the National Comprehensive Cancer Network (NCCN) provides guidelines for patients based on the NCCN Guidelines for CRC Screening, which align with the ACG recommendations [11]. The European guidelines also address CRC screening and diagnosis, emphasizing the importance of early detection and prevention strategies [12]. This diversity in screening options has often led to confusion among healthcare providers and patients regarding the most appropriate approach.

However, in 2018, the American Cancer Society made a fundamental update to its guidelines by giving a qualified recommendation to lower the screening age to begin at 45. This change was prompted by the rising incidence of colon and rectal cancers in individuals younger than 50, a trend that had been observed for over a decade. Modeling studies using two major models, Microsimulation Screening Analysis-Colon (MISCAN-Colon) and Colorectal Cancer Screening Intervention for Malaysia (CRC-SIM), informed this decision by projecting the potential impact of starting screening at age 45 versus 50, considering various screening modalities and their effectiveness in terms of life years gained [13,14].

In response to rising cases among the young population, the American College of Gastroenterology (ACG) updated its evidence-based screening guidelines for CRC in March 2021 [15]. The updated guidelines recommend that average-risk individuals begin CRC screening at age 45, aligning with previous recommendations for African Americans since 2005. The primary screening modalities recommended by the ACG include colonoscopy and the fecal immunochemical test (FIT), with other options such as the multitarget stool DNA test, CT colonography, and colon capsule also available. This recommendation aimed to mitigate the rising incidence of advanced adenomas, CRC, and ultimately, mortality from CRC, reflecting a paradigm shift in CRC screening practices to adapt to the changing epidemiology of the disease.

Despite their importance, CRC screening rates declined during the COVID-19 pandemic, adding to the challenges already faced in achieving optimal screening utilization [16]. The ACG updates in 2021 emphasize optimizing screening rates to achieve the aspirational target of over 80% to combat CRC effectively.

In conclusion, early CRC screening is essential for timely detection and prevention. By following evidence-based guidelines, we can reduce the burden of CRC and improve overall health outcomes. Regular updates to screening guidelines reflect advances in screening technology and our understanding of the disease, ensuring that healthcare providers have access to the most up-to-date information to guide their screening practices.

## 3. Advancements and Challenges in Colorectal Cancer Screening Guidelines

Guidelines encompass recommendations spanning various facets of CRC, including the patient population under consideration, risk assessment, screening tests, strategies, recommended starting and stopping ages, screening intervals, treatment or interventions, and screening for CRC in disadvantaged populations [17]. Dr. Shaukat explains how these recommendations are graded, distinguishing between strong ones and conditional suggestions, ensuring the guidelines are reliable [2].

CRC screening employs a variety of testing methods, including stool-based tests like gFOBT, FIT, and sDNA-FIT, which detect blood or cancer DNA in stool. Direct visualization tests such as colonoscopy, flexible sigmoidoscopy, CT colonography, and capsule endoscopy allow for polyp identification, aiding in CRC prevention [16]. Emerging techniques like blood-based biomarker tests, such as methylated SEPT9 DNA detection, and molecular imaging are being explored for CRC screening [3]. These methods provide clinicians with diverse tools to effectively detect and manage colorectal malignancies. Due to a lack of sufficient evidence of efficacy, the USPSTF recommendation does not include serum tests, urine tests, or capsule endoscopy for CRC screening [16].

In the latest guidelines, as anticipated, we find a significant modification is the reduction of the starting age for screening from 50 years to 45 years. This adjustment is projected to prevent approximately two to three cases of CRC and avert around one additional death [2]. Dr. Shaukat, in her presentation, highlights the various challenges associated with the new guidelines, including the potential harms of early screening, the significant burden on the healthcare system, resource accessibility, and ensuring equitable distribution of benefits across all socioeconomic classes and geographic regions [2]. She emphasizes how crucial family history is in CRC guidelines, identifying individuals at double the risk. Risk is influenced by factors like age, relatives’ age at diagnosis, relation degree, and number of affected relatives [18]. More affected relatives mean a higher CRC risk. For those with one first-degree relative (FDR) with CRC, colonoscopy every 5 to 10 years from age 40 to 50, or 10 years earlier than the FDR’s diagnosis, is recommended [17]. Alternatively, fecal immunochemical testing every 1 to 2 years is an option. FDRs with non-advanced adenomas or second-degree relatives with CRC should follow standard screening guidelines [18].

As individuals age, the risks associated with invasive CRC screening modalities increase. Hence, a consensus has emerged to cease screening between the ages of 76 and 85, considering the balance between benefits and harms [2]. Invasive procedures like colonoscopy carry serious risks, including cardiopulmonary complications, bowel perforation, hemorrhage, infection, and post-polypectomy syndrome [19]. Additionally, the bowel preparation required for these procedures may exacerbate dehydration or electrolyte imbalances, particularly in older adults or those with comorbidities [19]. Consequently, it is crucial to carefully assess the appropriateness and potential risks of CRC screening in older individuals to ensure patient safety and well-being.

Dr. Shaukat underscores the significance of assuring individuals undergoing screening that the process is both efficient and carries minimal risk of complications [2]. Endoscopists have access to several quality indicators to monitor their performance, including bowel preparation quality, cecal intubation rate, withdrawal time, adenoma detection rate (ADR), and post-colonoscopy surveillance interval [20]. Meeting minimal targets for each indicator reduces CRC risk post-screening, highlighting the importance of adhering to these standards for optimal patient outcomes.

## 4. Implications of Lowering Colorectal Cancer Screening Age: Insights and Considerations

Lowering the screening age for adenoma detection, particularly in the context of CRC screening, holds profound implications for public health and healthcare management. Firstly, this strategy is anticipated to lead to a notable surge in adenoma detection rates. By initiating screening at younger ages, the likelihood of identifying adenomas, including precancerous lesions, in individuals who are otherwise asymptomatic increases significantly [20]. This early detection facilitates intervention at a stage where the disease is more amenable to treatment, thereby potentially reducing morbidity and mortality associated with CRC. Moreover, lowering the screening age is expected to precipitate a discernible shift in the age distribution of adenoma detection. Traditionally, CRC screening primarily targets older individuals due to the higher incidence rates in this demographic [21]. However, by broadening the scope to include younger age groups, healthcare systems may need to reallocate resources and adapt planning strategies to accommodate the changing epidemiological landscape. This shift may also necessitate adjustments in healthcare policies and guidelines to optimize screening effectiveness across different age cohorts.

High-quality colonoscopy aims to reduce the incidence of CRC via the detection and removal of adenomas [22]. The adenoma detection rate (ADR) is defined as the proportion of patients undergoing a screening examination in which one or more histologically confirmed colorectal adenomas or adenocarcinomas were detected [23].

The largest determinant of adenoma and CRC risk is age. The minimum benchmark for screening colonoscopy in men and women 50 years old is 30% and 20%, with an overall ADR benchmark of 25%. The prevalence of adenoma increases with age. Reports show that adults younger than 50 years of age have a similar prevalence to that of individuals 50 and older [22]. To be more precise, the ADR and adenomas per colonoscopy (APC) are lower in the age group 45–49, although the risk of adenoma doubles from age 50–54 to age 70–74 with a similar increase for both men and women [24]. The studies undertaken had their limitations due to lack of consideration of race and ethnicity [25]. In conclusion, the studies found that ADR and APC in the 45–49 age group were comparable to those in the 50–54 group. Thus, lowering the age for screening is unlikely to change the ADR significantly, unless there is a drastic shift in the age distribution to younger individuals [26] (Table 1).

## 5. Future Directions: Evolving Frontiers in Colorectal Cancer Screening

Traditionally, CRC and other different malignancies have only been detectable in the blood when they are large and at an advanced stage. However, in recent years, various new techniques have emerged, with blood-based screening tests being one of them. This innovative screening technique can utilize cfDNA, proteomics, and metabolomics, allowing for the detection of tumor progression much earlier than expected during its pathogenesis [24].

Concurrently, AI and machine learning algorithms are being further employed to leverage their strength in the accurate differentiation between normal and cancerous cells. All these advancements have brought blood-based cancer diagnosis to the forefront of CRC screening research [25].

Medicare, as of now, mandates blood-based tests to meet specific criteria for national coverage, which are as follows: sensitivity ≥74%, specificity ≥90%, approval from the FDA, and inclusion as a recommended screening test in at least one professional society guideline [27]. Yet another important blood-based screening test is Septin-9, also known as Methylated Septin-9, which has shown strong promise as a screening option in the patient population unwilling to undergo stool-based or colonoscopy screening for CRC. Currently, it is not a frontline screening test due to its lower sensitivity and specificity when compared to other, conventional blood-based tests [28]. However, recent iterations and developments have shown promising improvements, with a reported sensitivity of 74% and a specificity of 87%, nearing the Medicare threshold.

There are currently various clinical trials happening using blood-based tests, some examples being Freenome, Guardant, CancerSEEK, with many more. These clinical trials are either recruiting or completing the recruitment phase, providing hope for the future of blood-based CRC screening [3]. Notably, in recent years, there has been the development of a multicancer early detection test, named GRAIL. In an ongoing validation prospective study with 6600 participants, it has a reported sensitivity of 51.5% and specificity of 99.5%. These results show promise, especially for the early detection of various solid tumors, such as colorectal, breast, pancreatic, and lung tumors, and also hematologic malignancies [29]. However, the lack of coverage by insurance and an out-of-pocket price of $949 have raised many concerns over its accessibility.

There is present a gradual adaptation of the miRNome in response to a specific dietary regimen, whether vegan, vegetarian, or omnivore diet, probably as a consequence of the epigenetic and metagenomic changes occurring in the gut, which are then reflected in stool. Tarallo et al. report two stool DEmiRNAs (miR-636 and miR-4739) which showed a downregulation with the increased duration of a vegan or vegetarian diet, independent of age, and which are also implicated in weight loss. miR-4739 has also been associated with the induction of adipogenic differentiation of human bone marrow stromal cells by targeting low-density lipoprotein receptor-related protein 3 (LRP3) [30]).

miR-143 and miR-145 are some other well-known tumor suppressor miRNAs, and low levels of miR-143-3p have been observed in stool of patients with CRC. Their upregulation in stool of vegan and vegetarian diets supports the lower cancer risk observed in relation to these dietary habits. The miR-143/miR-145 cluster has an essential role in intestinal epithelium regeneration by modulating the insulin growth factor signaling pathway, and it is also implicated in obesity [31]. miR-181 was also observed upregulated in the postprandial period of a high-saturated fat meal in peripheral blood mononuclear cells of healthy individuals [30].

Recent data report the relation between miRNAs in the fecal matter and their role in obesity and also CRC development. The comprehensive assessment of fecal miRNA and gut microbiome signatures might provide future insights into the development of more accurate personalized dietary patterns, recommendations aimed at the prevention/treatment of chronic diseases with significant clinical implications in CRC screening which may contribute to increased survival, and increased agreeability of a large subset of the population to screening [32].

## 6. Conclusions

Acknowledging the escalating prevalence of advanced malignancies and CRC among younger populations, notably in the 45–49 years age group, is fundamental for clinicians, insurance companies, and governmental health bodies. Statistical evidence reveals a concerning trend, with the prevalence rate of neoplasia in 45–49-year-olds closely mirroring that of 50–54-year-olds, showcasing a mere 0.5% reduction and remaining substantially lower than those aged 55 and older. Notably, the adenoma detection rate (ADR) registers 3–7% lower for individuals aged 50–55 compared to their older counterparts.

The advancements and challenges discussed in our article have significant clinical implications for healthcare providers and patients alike. The rising incidence of colorectal cancer (CRC) among individuals under 50 years old necessitates early detection and intervention. Lowering the CRC screening age to 45 for average-risk individuals, as recommended by the American Cancer Society (ACS) and other leading organizations, could lead to earlier detection of CRC, particularly in younger populations where incidence rates are rising, potentially reducing CRC-related morbidity and mortality. The shift in screening practices to lower the adenoma detection screening age may lead to a surge in adenoma detection rates. Early identification and removal of adenomas in asymptomatic younger patients could prevent their progression to CRC, thereby reducing the disease burden.

The expanding pool of at-risk individuals underscores the urgent need to broaden CRC screening approaches and improve adherence to regular screening protocols. Overcoming barriers such as lack of awareness, access disparities, and socio-economic factors is therefore paramount in ensuring equitable screening uptake across all demographics.

In this context, blood-based tests emerge as a crucial tool for early cancer detection, offering a non-invasive and accessible screening option. The emergence of blood-based tests, such as Septin-9 and other innovative technologies, offers a promising avenue for improving early CRC detection. These non-invasive tests, which leverage innovative techniques and AI algorithms, could detect tumors at a significantly earlier stage than traditional methods, enhancing screening rates and reducing the burden of CRC on healthcare systems.

The changing landscape of CRC epidemiology necessitates adaptations in healthcare systems, including reallocation of resources, updating of policies and guidelines, and addressing challenges related to accessibility and affordability of screening options. Healthcare providers must stay abreast of the latest guidelines and technologies to ensure early detection and effective management of CRC. Additionally, ensuring patient safety and quality assurance is crucial, particularly with the potential risks associated with invasive CRC screening modalities increasing with age. Therefore, careful assessment of the appropriateness and potential risks of CRC screening in older individuals is crucial to ensuring patient safety and well-being. Adherence to quality indicators such as bowel preparation quality, cecal intubation rate, withdrawal time, and adenoma detection rate can reduce post-screening CRC risk.

Looking ahead, the anticipation of future advancements in CRC screening seems close, with ongoing research poised to yield significant breakthroughs in the coming years. Beyond the immediate horizon, continued investment in innovative technologies, evolving screening guidelines, and efforts to improve accessibility and affordability will be pivotal in driving progress toward reducing the incidence and mortality of CRC.

In closing, we wish to support Dr. Shaukat’s suggestions and heed this call to action, urging stakeholders across the healthcare landscape to dedicate proper attention to CRC screening and commit to collaborative efforts aimed at advancing early detection strategies. By embracing these innovations and implementing them in clinical practice, providers can make a tangible difference in reducing CRC-related morbidity and mortality. By harnessing the power of innovation and collective action, we can confront the challenges posed by CRC head-on, ultimately saving lives and improving the well-being of individuals at a global level.

## Figures and Tables

**Table 1:** Comparison of ADR, APC, and detection rate of advanced neoplasia by age group and sex, with 95% CI.

	45–49 Y N = 4841	50–54 Y N = 58,914	*p* Value (Compared with 45–49)	50–75 Y N = 159,817	*p* Value (Compared with 45–49)
overall ADR	28.4% (27.1–29.6%)	31.1% (30.7–31.4%)	<0.001	35.6% (35.4–35.8%)	<0.001
ADR in men	34.8% (32.9–36.8%)	38.3% (37.7–38.9%)	<0.001	43.0% (42.6–43.3%)	<0.001
ADR in women	22.6% (21.0–22.4%)	24.4% (23.9–24.9%)	0.001	29.0% (28.7–29.3%)	<0.001
APC	0.44 (0.41–0.46%)	0.49 (0.48–0.49%)	<0.001	0.59 (0.58–0.59%)	<0.001

## Data Availability

Data are contained within the article.
